# Bohler’s angle’s role in assessing the injury severity and functional outcome of internal fixation for displaced intra-articular calcaneal fractures: a retrospective study

**DOI:** 10.1186/1471-2482-13-40

**Published:** 2013-09-24

**Authors:** Yanling Su, Wei Chen, Tao Zhang, Xingwang Wu, Zhanpo Wu, Yingze Zhang

**Affiliations:** 1 Department of Orthopaedic Surgery, the Third Hospital of Hebei Medical University, Shijiazhuang, Hebei 050051, PR China

**Keywords:** Calcaneus, Displaced intra-articular fracture, Böhler’s angle, Functional outcome, Sanders classification

## Abstract

**Background:**

Controversy exits over the role of Böhler’s angle in assessing the injury severity of displaced intra-articular calcaneal fractures and predicting the functional outcome following internal fixation. This study aims to investigate whether a correlation exists between Böhler’s angle and the injury severity of displaced calcaneal fractures, and between surgical improvement of Böhler’s angle and functional outcome.

**Methods:**

Patients treated operatively for unilateral closed displaced intra-articular calcaneal fractures from January 1, 2004 to March 31, 2008 were identified. The Böhler’s angles of both calcaneus were measured, and the measurement of the uninjured foot was used as its normal control. The difference in the value of Böhler’s angle measured preoperatively or postoperatively between the angle of the injured foot and that of the contralateral calcaneus were calculated, respectively. The change in Böhler’s angle by ratio was calculated by dividing the difference value of Böhler’s angle between bilateral calcaneus by its normal control. The injury severity was assessed according to Sanders classification. The functional outcomes were assessed using American Orthopaedic Foot & Ankle Society hindfoot scores.

**Results:**

274 patients were included into the study with a mean follow-up duration of 71 months. According to Sanders classification, the fracture pattern included 105 type II, 121 type III and 48 type IV fractures. According to American Orthopaedic Foot & Ankle Society hindfoot scoring system, the excellent, good, fair and poor results were achieved in 104, 132, 27, and 11 patients, respectively. The preoperative Böhler’s angle, difference value of Böhler’s angle between bilateral calcaneus, and change in Böhler’s angle by ratio each has a significant correlation with Sanders classification (rs=−0.178, *P*=0.003; rs=−0.174, *P*=0.004; rs=−0.172, *P*=0.005, respectively), however, is not correlated with functional outcome individually. The three postoperative measurements were all found to have a significant correlation with American Orthopaedic Foot & Ankle Society hindfoot scores (rs=0.223, *P*<0.001; rs=0.224, *P*<0.001; rs=0.220, *P*<0.001, respectively). However, these correlations were all weak to low.

**Conclusions:**

There was a significant correlation between preoperative Böhler’s angle and the injury severity of displaced intra-articular calcaneal fractures, but only postoperative Böhler’s angle parameters were found to have a significant correlation with the functional recovery.

## Background

Displaced intra-articular calcaneal fractures constitute 70% of all calcaneus fractures in adults [1-4]. Böhler’s angle is frequently assessed when evaluating this type of fractures on lateral plain X-ray films [5]. A number of studies have examined the correlation between Böhler’s angle and the functional outcome of displaced intra-articular calcaneal fractures, but yielded mixed results [6-14]. Some studies suggest that surgical restoration of Böhler’s angle improves the outcome, and has a prognosis value of postoperative success as well as predictive value for subtalar joint fusion [6-12]. Some indicate the initial Böhler’s angle is highly prognostic regardless of treatment modality [13]. However, Ibrahim et al. reported no correlation at all between Böhler’s angle and functional outcome based upon the assessment of their 15-year follow up of a randomized controlled trial of displaced calcaneal fractures treated conservatively versus operatively [14]. The controversy may be partially due to the limitations of original study design and the comparatively small sample size. Böhler’s angle varies little between left and right foot of an individual [13], but considerably among individuals from 25 to 40 degrees. Given the variability of Böhler’s angle between different individuals, we think it is an objective way to obtain the Böhler’s angle of uninjured foot as the patient’s own normal control to assess the correlation between Böhler’s angle and the functional outcome. Accordingly, we propose a novel way to describe Böhler’s angle with the uninjured foot as the individual own reference. This study will address the following questions: (1) whether there is a correlation between Böhler’s angle and the injury severity of displaced intra-articular calcaneal fractures; (2) Whether surgical improvement of Böhler’s angle is related to the functional outcome; and (3) To what degree Böhler’s angle need to be improved to achieve a satisfactory outcome if such correlation exists.

## Methods

Between January 1, 2004 and March 31, 2008, 972 displaced intra-articular calcaneal fractures in 896 patients who were treated operatively in our department were identified through the trauma registration system. The inclusion criteria were: aged 18 years and above; closed unilateral fractures; treated with surgical internal fixation; absence of polytrauma of the injured foot; computed tomography (CT) scans performed on the injured calcanues; and absence of severe medical ailments. Patients with other concurrent fractures in both lower extremities were excluded.

### Surgical technique and postoperative management

In the Orthopedic department of our hospital, only two surgical methods were applied to treat displaced intra-articular calcaneal fractures during the study period: (1) the traditional L-shaped extended lateral approach with fractures fixed by plate and screws, (2) a minimally invasive approach featured an anatomic plate and multiple compression bolts. Operation was performed on an average of 5.7 days (range, 3–13 days) after injury, when swelling subsided and positive wrinkle appeared on the hindfoot. The surgery aimed to restore the width, length and height of the calcaneus, Böhler angle, the alignment of the heel bone and the posterior articular surface with step off <3 mm.

### Minimally invasive fixation technique

As described in previous reports [15,16], this minimally invasive procedure started with transverse insertion of one or two Steinmann pins into calcaneal tuberosity, followed by distraction of pins to restore primarily the length and height of the calcaneus. Another one or two Steinmann pins were introduced forward across the fracture line for repeated percutaneous leverage to reduce the articular surface and the height of the calcaneus, which can help restore Böhler angle to normal range. If satisfactory reduction was obtained, the Steinmann pins were drilled forward to the anterior part of calcaneus for provisional fixation. Then, a 3.5-cm longitudinal incision was made on the posterior part of lateral hindfoot, and a subcutaneous tunnel was created with a periosteal elevator. The anatomical plate of appropriate size was inserted into the tunnel, and then fixed with two to four (usually three) compression bolts under intraoperative fluoroscopic control. The nut was tightened on each screw, and integrated with the anatomical plate as a whole, which produced tremendous compressive force to reduce the width and height of the calcaneus to the utmost extent. The distal part of the compression bolt was broken off at the constricted area. In cases of severe collapse, or displaced, involving the center of articular surface, a small lateral incision through the sinus tarsal could be made to get direct access to the articular surface for an anatomical reduction. Sometimes, additional cancellous screws were used to secure the multi-fragment fractures.

### Open reduction and internal fixation technique

The L-shaped extended lateral approach was made. The peroneal tendons and calcaneofibular ligament were retracted to expose the fracture. The posterior facet was elevated and the joint was reduced under direct visualization. The Kirschner wires were used for temporary fixation, followed by definitive fixation with a plate and screws. In case of significant bone defect, the iliac bone graft was used to fill up the defect.

### Postoperative management

All patients completed the same standardized postoperative rehabilitation protocol. The injured feet were not casted or placed in a removable boot after operation. Patients were encouraged to start non-weight-bearing exercise, including extension and planter flexion of toes, ankle, and subtalar joints as soon as pain can be tolerated. Crutch-assisted walking was allowed two or three days postoperatively. The amount of time for non-weight-bearing was 8 weeks after operation as recommended by Schepers et al. [17]. Partial weight bearing was started after 8 weeks and advanced to full weight bearing, which was allowed with radiographic evidence of fracture union, generally at three months postoperatively. All patients were routinely followed up at 6 weeks, 3, 6 and 12 months after operation and then yearly. Functional outcomes were assessed based upon American Orthopaedic Foot & Ankle Society hindfoot score at the final follow-up.

### Data collection and statistical analysis

**Figure 1:**
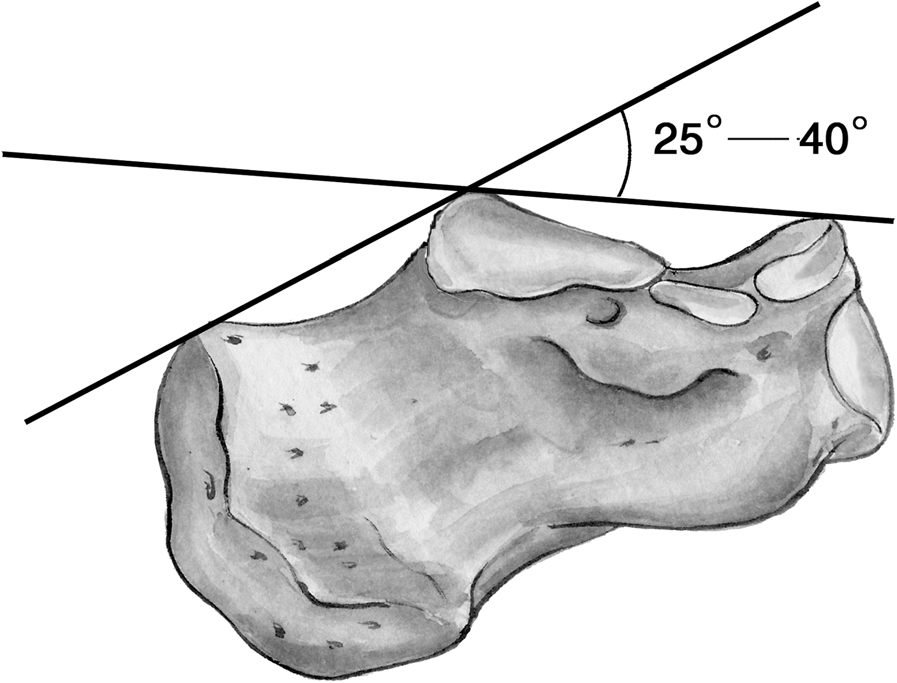
**The schematic drawing of the modality of measurement of Böhler’s angle.** Böhler’s angle can be measured with the use of two intersecting lines: one drawn from anterior process of the calcaneus to the highest part of posterior articular surface and a second drawn from the same point of posterior articular surface to the most superior point of tuberosity.

The severity of calcaneal fractures were assessed according to Sanders classification determined by CT scans. The radiographs of both feet for each patient were collected. The Böhler’s angles of both calcaneus were measured (Figure 1) with MB-ruler on the PACS workstation. Each measurement was taken twice by two trained research assistants and the final measurement was the average of the two. Any discrepancy was settled by consensus. The difference value between Böhler’s angle measured preoperatively or postoperatively on the injured foot and that of the contralateral unaffected calcaneus was calculated, respectively. The preoperative change in Böhler’s angle by ratio was calculated by dividing preoperative difference value of Böhler’s angle between bilateral calcaneus by Böhler’s angle of its normal control. Similarly, the extent of surgical restoration of Böhler’s angle by ratio is obtained by dividing postoperative difference value of Böhler’s angle between bilateral calcaneus by its normal control.

The data were analyzed with SPSS 13.0 for Windows (SPSS Inc., Chicago, IL, USA). Categorical variables were recorded as numbers and percentiles with frequency tables. Continuous variables were expressed as mean±standard deviation (SD). The Mann–Whitney *U* test was applied to analyze continuous variables. The correlations between Böhler’s angle and American Orthopaedic Foot & Ankle Society hindfoot scores, Böhler’s angle and Sanders classification, American Orthopaedic Foot & Ankle Society hindfoot scores and Sanders classification were analyzed with bivariate correlation (spearman’s rank correlation). To interpret Spearman, value of rs of 0.9 to 1 indicates very strong correlation; between 0.7 and 0.89, strong correlation; between 0.5 and 0.69, moderate; between 0.3 and 0.49, moderate to low; between 0.16 and 0.29, weak to low; and below 0.16, correlation is too low to be meaningful [18]. A *P* value less than 0.05 was regarded as statistically significant.

The study has been approved by the review board of the Third Hospital of Hebei Medical University. The investigations have been conducted according to the principles expressed in the Declaration of Helsinki. Signed informed consents have been obtained from all patients.

## Results

328 patients with 328 displaced intra-articular calcaneal fractures met the inclusion criteria. Among them, 274 patients were available for follow up with a mean duration of 71 months (range, 48–99 months). There were 231 males and 43 females, with an average age of 38.5 years (range, 18–69 years). The injury mechanisms included a fall from a height in 145 patients, a traffic injury in 62, a crush injury in 51, and a sprain in 16. According to Sanders classification, the fracture pattern included 105 type II, 121 type III and 48 type IV fractures. According to American Orthopaedic Foot & Ankle Society hindfoot score, the functional outcomes were excellent in 104 patients (37.96%), good in 132 (48.18%), fair in 27 (9.85%), and poor in 11 (4.01%) (Table 1). Soft tissue complications were reported in 32 patients, including 13 patients with superficial infection, 5 wound edge necrosis, 2 deep infection, 3 sural nerve injury, 4 medial plantar nerve injury, and 7 with restricted movement of flexor hallucis longus tendons (2 with concurrent medial plantar nerve injury). Superficial infection and wound edge necrosis were resolved by dressing changes. Deep infection extended to the level of hardware in both patients, which required hardware removal. Hardware removal and neurolysis was done on 2 patients with sural nerve injury at 12 weeks after operation, and marked symptom relieve was achieved postoperatively. Five out of the seven patients with restricted movement of flexor hallucis longus tendons had pain and tenderness due to the compression bolts passing above the tendons. Hardware was removed and the symptoms were alleviated significantly.

**Table 1:** The American Orthopaedic Foot & Ankle Society scores and Sanders classification

**Sanders classification**	**American Orthopaedic Foot & Ankle Society score**			
	**Excellent (n, %)**	**Good (n, %)**	**Fair (n, %)**	**Poor (n, %)**
**II**	55	45	4	1
**III**	43	64	9	5
**IV**	6	23	14	5
**Total**	104 (37.96)	132 (48.18)	27 (9.85)	11 (4.01)

The pre- and post- operative lateral X-ray films of the injured feet were collected in all 274 patients. The X-ray films of the uninjured feet were taken in 106 patients preoperatively and in 168 patients at follow-ups. The average values of the three preoperative measurements (Böhler’s angle, difference value of Böhler’s angle between bilateral calcaneus and change in Böhler’s angle by ratio) were 6.36 degrees (range, -30-32 degrees), -25.42 degrees (range, -62-0 degrees), and −0.80 (range, -1.94-0), respectively. The average values of the three postoperative measurements (Böhler’s angle, difference value of Böhler’s angle between bilateral calcaneus and change in Böhler’s angle by ratio) were 28.17 degrees (range, 0–60 degrees), -3.62 degrees (range, -28-22 degrees), and −0.10 (range, -1-0.58), respectively. For patients who achieved excellent or good results based on American Orthopaedic Foot & Ankle Society hindfoot score, the three postoperative measurements were 28.89±7.24 degrees (range, 9–46 degrees), -2.83±8.05 degrees (range, -23-18 degrees), and 0.08±0.26 (range, -0.72-0.82), respectively.

The three preoperative measurements were all found to have a significant correlation with Sanders classification (rs=−0.178, *P*=0.003; rs=−0.174, *P*=0.004; rs=−0.172, *P*=0.005, respectively) (Table 2). Interestingly, we found that none of the three preoperative measurements was correlated with American Orthopaedic Foot & Ankle Society hindfoot score (rs=0.086, *P*=0.156; rs=0.085, *P*=0.161; rs=0.100, *P*=0.101, respectively). The three postoperative measurements were all found to have a significant correlation with American Orthopaedic Foot & Ankle Society hindfoot score (rs=0.223, *P*<0.001; rs=0.224, *P*<0.001; rs=0.220, *P*<0.001, respectively) (Table 2). Of the four subjective evaluation categories in American Orthopaedic Foot & Ankle Society hindfoot scores, postoperative Böhler’s angle were found to correlate with pain, walking distance and walking surface (rs=0.259, *P*=0.003; rs=0.177, *P*=0.045; rs=0.196, *P*=0.027, respectively), but not correlate with the category of activity limitation. No correlation was found between any of the four subjective variables and the difference value of Böhler’s angle between bilateral calcaneus or the change in Böhler’s angle by ratio (Table 3). Because the rs value ranged from −0.172 to 0.259, it should pointed out that the above-mentioned correlations were weak to low.

**Table 2:** The correlation between Böhler’s angle, the difference value of Böhler’s angle between bilateral calcaneus, or the change in Böhler’s angle by ratio and the injury severity or functional outcomes

	**Correlation**		**rs-value**	***P*-Value**
Preoperative	Böhler’s angle	Sanders classification	−0.178	0.003
	The difference value of Böhler’s angle between bilateral calcaneus	Sanders classification	−0.174	0.004
	The change in Böhler’s angle by ratio	Sanders classification	−0.172	0.005
Preoperative	Böhler’s angle	Functional outcome	0.086	0.156
	The difference value of Böhler’s angle between bilateral calcaneus	Functional outcome	0.085	0.161
	The change in Böhler’s angle by ratio	Functional outcome	0.100	0.101
Postoperative	Böhler’s angle	Functional outcome	0.223	<0.001
	The difference value of Böhler’s angle between bilateral calcaneus	Functional outcome	0.224	<0.001
	The change in Böhler’s angle by ratio	Functional outcome	0.220	<0.001

Note: The functional outcomes were assessed using American Orthopaedic Foot & Ankle Society scores.

**Table 3:** The correlation between the four subjective variables of American Orthopaedic Foot & Ankle Society scores and the postoperative Böhler’s angle, difference value of Böhler’s angle between bilateral calcaneus or change in Böhler’s angle by ratio

**Correlation**		**rs-value**	***P*-Value**
**Pain**	Böhler’s angle	0.259	0.003
	The difference value of Böhler’s angle between bilateral calcaneus	0.153	0.084
	The change in Böhler’s angle by ratio	0.169	0.052
**Activity limitation**	Böhler’s angle	0.058	0.513
	The difference value of Böhler’s angle between bilateral calcaneus	0.013	0.882
	The change in Böhler’s angle by ratio	0.020	0.827
**Walking distance**	Böhler’s angle	0.177	0.045
	The difference value of Böhler’s angle between bilateral calcaneus	0.084	0.342
	The change in Böhler’s angle by ratio	0.128	0.152
**Walking surface**	Böhler’s angle	0.196	0.027
	The difference value of Böhler’s angle between bilateral calcaneus	0.061	0.491
	The change in Böhler’s angle by ratio	0.092	0.302

The correlation between American Orthopaedic Foot & Ankle Society hindfoot score and Sanders classification was also analyzed, and a significant negative correlation was identified (rs=−0.362, *P*<0.001). The correlation was moderate to low.

## Discussion

Based upon the comprehensive statistic analysis of the data obtained from 274 patients, we found that the preoperative Böhler’s angle, difference value of Böhler’s angle between bilateral calcaneus, and change in Böhler’s angle by ratio each has a significant positive correlation with Sanders classification, while the three postoperative measurements each also has a significant positive correlation with American Orthopaedic Foot & Ankle Society hindfoot score. To some extent, the preoperative Böhler’s angle can indicate the injury severity of displaced intra-articular calcaneal fractures, while the postoperative angle can provide prognostic information with regard to the functional outcomes.

Various radiographic parameters have been used to describe calcaneal fractures. Böhler’s angle is an accepted method of quantifying fracture displacement and has a prognostic value in predicting morbidity associated with calcaneal fractures [5,13]. Mitchell et al. reported that there was a strong association between Böhler’s angle and Sanders classification based upon the analysis of the data obtained from 80 patients [19]. However, we identified a weak to low correlation between preoperative Böhler’s angle and Sanders classification after analyzing the data obtained from 274 patients. Similarly, the preoperative difference value of Böhler’s angle between bilateral calcaneus and the change in Böhler’s angle by ratio were also found to significantly correlate with Sanders classification. Our findings indicated that there was no correlation between functional outcomes and preoperative Böhler’s angle, difference value of Böhler’s angle between bilateral calcaneus or change in Böhler’s angle by ratio. However, Loucks et al. reported that extreme diminution of Böhler’s angle of the injured feet represented a significantly diminished outcome at two-year follow ups [13]. Buckley et al. considered Böhler’s angle as a surrogate measure of the amount of energy absorbed by the foot [20]. That is to say, the greater the energy absorbed by the calcaneus, more severe the fractures. The extreme diminution of preoperative Böhler’s angle indicated more severe injury of displaced calcaneal fractures. Technically, it is more difficult to restore Böhler's angle of severely displaced calcaneal fractures to normal range than those with less severe injuries. Therefore, theoretically, those severely injured cases would be more likely to suffer poor outcome. Our findings that there was a significant negative correlation between American Orthopaedic Foot & Ankle Society hindfoot scores and Sanders classification (rs=−0.362, *P*<0.001) also support this hypothesis.

Although the optimal treatment of displaced intra-articular calcaneal fractures continue to elude investigators [21,22], open or close reduction and internal fixation has gained its popularity for the ability to restore morphology of the calcaneus and articular congruity. Therefore, only surgically treated patients were included into this study. Pozo et al. reported that although two-thirds of patients with calcaneal fractures reached a point of maximal functional recovery at two to three years, 24% continued to improve for six years [23]. Therefore, to identify whether surgical improvement of Böhler’s angle is correlated with the maximal functional outcomes, we enrolled patients treated operatively from January 2004 to March 2008 into this study, who were followed up with a mean duration of 71 months.

The Böhler’s angle can be used to guide the fracture reduction intraoperatively. Restoring the Böhler’s angle back to normal range of 25–40 degrees is one of the surgical goals in clinical practice [15], which is one of the important factors to obtain satisfactory results [24,25]. However, study findings over whether restoration of Böhler’s angle correlates with an improved clinical outcome have been mixed. There are two opposing viewpoints among the published literature (Table 4). Some authors think that surgical restoration of Böhler’s angle can improve the functional outcome of the injured feet [6-10], and that Böhler’s angle is of prognostic relevance [26-29]. In a review of 70 cases with displaced intra-articular calcaneal fractures, Paul et al. reported that patients were found to have a good outcome following operative treatment when Böhler’s angle was optimally restored >10 degrees [25]. Slightly different from Paul’s study, Buckley et al. reported a markedly good functional outcome at a long-term follow ups in patients with restored Böhler’s angle >15 degrees in both operative and conservative group [20]. In Makki’s study, restoration of Böhler’s angle ≥30 degrees was associated with a better outcome based upon the data of 47 patients [24]. And vice versa, poor functional outcome can be seen in patients without restoration of Böhler’s angle. Janzen et al. reported that a loss of Böhler’s angle measured at follow ups was associated with a poor clinical outcome [30]. Paley and Hall [31] found that the ratio of Böhler’s angle of the injured side to the normal side was significantly lower in patients with unsatisfactory outcomes and concluded that a decrease in this ratio was a negative prognostic factor. However, Hutchinson, Kundel, Ibrahim and Mauffrey held the opposite opinion that there was no correlation at all between Böhler’s angle measured at follow-ups and the final functional outcomes in neither operative nor conservative group [14,22,26,32]. More interestingly, Loucks et al. reported that although Böhler’s angle increased after open reduction and internal fixation, the clinical outcome diminished [13]. In their study, a statistically significant negative correlation (rs=−0.300; *p*<0.05) was found between the change in angle (angle measured at 3-month follow-up minus angle at the time of injury) and the SF-36 score at two-year follow up in a surgical treatment group.

**Table 4:** Summarization of the published articles and the current study regarding the role of Böhler’s angle in assessing the injury severity and functional outcome for displaced intra-articular calcaneal fracture

**No.**	**Cases (n)**	**Treatment (n)**		**Follow up (year, average (range))**	**Correlation**
		**Operation**	**Conservation**		
**1**	45	33	18	4.5 (1–11)	A loss of Böhler’s angle measured at follow ups was associated with a poor clinical outcome [27].
**2**	44	52	0	4-14	The ratio of Böhler’s angle of the fractured side to that of the normal side was significantly lower in patients with unsatisfactory outcomes at final follow up evaluation [28].
**3**	43	47	0	1	Böhler’s angle measured at the final follow-up did not correlate with the clinical result [20].
**4**	63	30	33	5.1	No correlation between Böhler’s angle and final functional outcomes [23].
**5**	88	44	44	2	There was a significant negative correlation between change in Böhler’s angle (angel measured at 3-month minus angle at the time of injury) and SF-36 score in surgical group [13].
**6**	70	29	41	6.5 (4–15)	Patients with displaced fractures had a good outcome following operative treatment with restored Böhler’s angle>10 degrees [22].
**7**	26	15	11	15 (11–18)	No correlation between Böhler’s angle and functional outcome [14].
**8**	16	16	0	2 (1–4)	No correlation between good restoration of Böhler’s angle and high functional score [29].
**9**	47	47	0	10 (7–15)	Restoration of Böhler’s angle ≥30 degrees was associated with a better outcome [21].
**10**	274	274	0	6 (4–8)	The preoperative Böhler’s angle has a significant correlation with Sanders classification. The postoperative Böhler’s angle has a significant correlation with the final functional outcome (The current study).

Our findings support the former viewpoint that postoperative Böhler’s angle can be used to predict the clinical outcomes. We found a significant positive correlation between postoperative Böhler’s angles and American Orthopaedic Foot & Ankle Society hindfoot scores. Böhler’s angle is defined by two intersecting lines: one drawn from anterior process of the calcaneus to the highest part of posterior articular surface and a second drawn from the same point of posterior articular surface to the most superior point of tuberosity. Böhler’s angle measures the height of posterior articular facet [33]. Anatomical reduction of posterior articular surfaces of the calcaneus is an important goal for the treatment of displaced intra-articular calcaneal fractures, which can help restore Böhler’s angle, predicting a promising prognosis. Our study indicated that restoration of Böhler’s angles≥9 degrees was associated with good to excellent results, similar to the findings reported by Paul et al. [25].

To investigate the most correlated pattern of Böhler’s angles with functional outcomes, we introduced the Böhler’s angle of uninjured foot as its normal control. The correlation between American Orthopaedic Foot & Ankle Society hindfoot scores and the postoperative difference value of Böhler’s angle between bilateral calcaneus and the change in Böhler’s angle by ratio was also analyzed, respectively. Different from our initial hypothesis, the two postoperative measurements were not superior to Böhler’s angle in predicting functional outcomes. In addition, postoperative Böhler’s angle was found to correlate with three out of the four subjective variables of American Orthopaedic Foot & Ankle Society hindfoot scores. However, no significant correlation was found between any of the four variables and postoperative difference value of Böhler’s angle between bilateral calcaneus or the change in Böhler’s angle by ratio.

In the current study, although significant correlations were identified both between postoperative Böhler’s angles and American Orthopaedic Foot & Ankle Society hindfoot scores and between preoperative angle and Sanders classification, the rs values ranged from −0.172 to 0.224, which indicates a weak to low correlation [18]. Studies with small sample sizes are more prone to errors or bias in patient selection and data collection than those with large sample sizes, which is more likely to compromise the results, and lead to the mixed findings as summarized in Table 4. To the best of our knowledge, our study included the largest patient sample size among the published studies to detect the role of Böhler’s angle in predicting the injury severity and functional recovery, and the comprehensive analysis of a large pool of data should be very helpful to settle this disputes.

The study has some limitations. It is limited by the retrospective nature of the study design. Various parameters may influence the functional outcomes of displaced intra-articular calcaneal fractures, such as the reduction quality of posterior articular surface and the reduced width and height of the calcaneus. As part of the study design, we did not include those factors. Another limitation is that taking the lateral radiographs of the contralateral unaffected feet increased the burden of radiation exposure for all patients.

## Conclusions

The preoperative Böhler’s angle has a significant correlation with the injury severity of displaced intra-articular calcaneal fractures. The postoperative Böhler’s angle has a significant positive role in predicting the functional recovery. Restoration of Böhler’s angle should be an important reduction index during surgical treatment of displaced intra-articular calcaneal fractures, and of ≥9 degrees is essential to achieve satisfactory functional outcomes.

## Competing interests

The authors declare that they have no competing interests.

## Authors’ contributions

YZ, YS and WC designed research; YS, WC and ZW made substantial contributions to acquire X-ray films and measured Böhler’s angle; TZ and XW analyzed CT images and conducted follow ups; TZ and XW analyzed data and performed statistical analysis; YS and WC drafted and designed the manuscript; YZ had primary responsibility for final content; All authors read and approved the final manuscript.

## Pre-publication history

The pre-publication history for this paper can be accessed here:

http://www.biomedcentral.com/1471-2482/13/40/prepub
